# Preferences of Hospital Pharmacists for the Different Attributes of Intravitreal Treatments for Neovascular Age-Related Macular Degeneration and Diabetic Macular Edema in Spain: The SEEKING Study

**DOI:** 10.3390/pharmacy13030068

**Published:** 2025-05-14

**Authors:** José Luis Poveda, Pablo Arnáiz, Silvia López, Belén Muñoz, Anxo Fernández-Ferreiro

**Affiliations:** 1 Hospital La Fé, 46026 Valencia, Spain; poveda_josand@gva.es; 2 Roche Farma S.A., 28042 Madrid, Spain; pablo.arnaiz@roche.com (P.A.); silvia.lopez.sl4@roche.com (S.L.); belen.munoz_molina@roche.com (B.M.); 3 Pharmacy Department, University Hospital of Santiago de Compostela (SERGAS), 15706 Santiago de Compostela, Spain; 4 FarmaCHUSLab, Health Research Institute Santiago Compostela (IDIS), 15706 Santiago de Compostela, Spain

**Keywords:** conjoint analysis, diabetic macular edema, hospital pharmacists, intravitreal treatment, neovascular age-related macular degeneration, preferences

## Abstract

The process of evaluation and selection of drugs in Spain is currently changing, with hospital pharmacists (HPs) having a growing relevance. This cross-sectional observational study aimed to assess HPs’ preferences for different hypothetical intravitreal treatments for neovascular age-related macular degeneration (nAMD) and diabetic macular edema (DME). Best corrected visual acuity (BCVA), ocular adverse events (AE), annual drug cost, available presentation, and mechanisms of action (MoA) were the selected attributes. A conjoint analysis was used. Ninety-one HPs completed the study. The mean (SD) age was 39.2 (10.2) years, 60.4% were female, and the mean (SD) time of experience as HP was 12.6 (8.3) years. For nAMD treatments, BCVA (38.6%) and ocular AE (27.3%) were the most important attributes, while annual drug cost (16.3%), available presentation (11.1%), and MoA (6.7%) were not as important. For DME drugs, BCVA (44.6%) and ocular AE (25.5%) were the most significant factors; annual drug cost (17.9%), the drug’s available presentation (7.3%), and MoA (4.8%) were not considered to be as crucial. Preferences were comparable independent of HP experience. Effectiveness and safety were the most important attributes when choosing a drug. Comprehending the significant characteristics for HPs could potentially improve their collaborative function within multidisciplinary teams involved in intravitreal treatments.

## 1. Introduction

It may be noted here that age-related macular degeneration (AMD) and diabetic macular edema (DME) are frequent retinal degenerative diseases and are responsible for the majority of cases of blindness due to retinal disease [1]. AMD and DME are expected to increase in prevalence in the coming years due to the aging of the population [2,3,4,5]. Thus, early diagnosis and rapid treatment onset are essential to achieve the best results and preserve vision [5,6,7].

AMD is a chronic, progressive, and severe disease characterized by degenerative alterations in the central retina, which constitutes the main cause of severe visual impairment and irreversible blindness in elderly people in developed countries [8,9]. The overall prevalence of AMD is estimated to be approximately 8.7% worldwide, although with substantial variations among different populations [5]. According to the World Health Organization (WHO), about 196 million people are affected around the world, with the overall incidence of AMD increasing from 4.2% in patients aged 45–49 years to 27.2% in those aged 80–85 years [10]. In the European Union, approximately 67 million people are currently affected by any form of AMD, and this number is expected to increase to 77 million by 2050 [4]. The most severe form of AMD is neovascular AMD (nAMD), responsible for 90% of the severe vision loss associated with AMD [8,11].

DME is a multifactorial and complex disease characterized by exudative fluid accumulation in the macula, and it is the main cause of blindness among patients with diabetes mellitus (DM) [12,13]. The prevalence of DME among individuals with DM in Europe was estimated to be 3.7%, with a mean annual incidence of 0.5% [3].

Currently, there are many treatments available for these retinopathies, mainly anti-vascular endothelial growth factor (anti-VEGF) agents for nAMD and anti-VEGF or corticosteroids for DME [14,15]. While the drug effectiveness remains the most valuable characteristics, treatment choice considers other aspects (safety, cost, etc.), and different stakeholders (patients, doctors, and hospital pharmacists [HP]) influence medical decision-making while assessing the treatment characteristics in a different way [16,17]. HP are especially relevant in selecting treatments for conditions such as intravitreal treatments and providing support to decision- making [17,18].

Elicitation of preferences is a method used to evaluate the significance that individuals attribute to various features of a specific product. This approach has been employed to comprehend the importance that stakeholders place on the diverse characteristics of medical interventions [19,20]. Preferences for elicitation using conjoint analysis (CA) techniques have been successfully applied to assess for preferences in different settings [18,21,22]. CA is a technique based on mathematical psychology and Lancaster’s theory of value that aims to identify the value that subjects assign to different attributes of a given product [23]. CA presents individuals with a series of hypothetical product profiles, each characterized by a set of attributes and levels for each attribute. Its rationale is that it closely resembles the everyday decisions individuals make when choosing between alternatives with multiple attributes, and it has been used to assess the preferences of different healthcare professionals [16,18,23,24].

Currently, in Spain, there is an ongoing paradigm shift in drug evaluation and selection. This shift is associated with the growing relevance of clinical decision-making based on values and the important role that HPs have in therapeutic choices. The current research intends to evaluate HPs’ preferences for different hypothetical intravitreal treatments for nAMD and DME, according to a set of selected attributes, to quantify the weight of each studied attribute. As a secondary objective, this study aims to assess whether the preferences are comparable at different levels of clinical experience.

## 2. Materials and Methods

### 2.1. Study Design and Participants

The SEEKING study is a cross-sectional observational study that aims to understand the HPs’ preferences among different attributes of hypothetical intravitreal treatments for nAMD and DME. HPs actively involved in the management of intravitreal treatments of patients with nAMD or DME were invited to participate in the study via email from July to October 2023. The study was approved by the Research Ethics Board of Hospital Clínico San Carlos (code number: 23/307-E). Due to the particular nature of the data collected (not patient data, but opinions/points of view of participants), and in compliance with regulatory requirements, HPs were informed about the study objectives and the confidentiality and preservation of their responses. Participants provided informed consent before the start of the study.

### 2.2. Variables

The present CA was not related to any medicinal product or treatment. No data from patients were collected either individually or collectively, and the study subjects were HPs, so only variables related to them were collected.

The CA evaluated eight hypothetical treatment scenarios with different combinations of attributes and levels of assessment, which were presented to the HPs to rank them according to their preferences. Two independent preferences exercises were presented, one for nAMD and one for DME, including the same attributes for each hypothetical treatment, but with different levels for each attribute. To determine the scenarios, two steps were carried out, as follows:

1. Selection of attributes and levels (Supplementary Table S1) [25]: Five treatment attributes, including best corrected visual acuity (BCVA), ocular adverse events (AE), annual drug cost, available presentation, and mechanism of action (MoA), were selected from those used in clinical trials and other studies, extracted from a literature review, and after consensus of an expert panel of HPs, and also determined by a consensus of the most relevant levels for each attribute.
2. Application of an orthogonal design with Statistical Package for the Social Sciences (SPSS): The five attributes with two levels each (nAMD) and the five attributes, four of them with two levels and one with three levels (DME) defined in step 1 (Supplementary Table S1), were used. The maximum number of possible scenarios was 32 for nAMD and 48 for DME. Since it is impossible to establish a ranking among all these possibilities at a cognitive level, eight scenarios (cards from A to H) were selected by applying an orthogonal design with SPSS. HPs’ preferences for the final eight scenarios were evaluated with a ranking system (Supplementary Table S2).

In addition, participants answered questions related to their sociodemographic and professional practice characteristics as secondary variables.

### 2.3. Analysis

The main analysis was focused on the preferences of HPs for several attributes of intravitreal treatments. Ordinary least squares (OLS) regression was selected in the current research to estimate parameters. From the part-worth, CA calculates the relative importance that individuals assign to the different attributes that make up each hypothetical treatment. Attribute importance is the difference between their highest and lowest utility levels. If the distance between the utility levels of an attribute is large, that attribute has a larger bearing on the respondents’ choice of treatment than another one where the distance is not as large. The relative importance of each attribute was obtained by dividing its individual importance by the sum of all. The most important attribute is the one whose levels are extreme in terms of utilities.

To assess if preferences were comparable among different levels of clinical experience, a preference analysis was divided into two groups depending on the distribution of years in managing intravitreal treatment. The categorization was established according to the median time obtained in the sample:

1. Some experience (≤5 years): Years of experience in managing intravitreal treatments for nAMD/DME equal to or less than the median time obtained in the study sample.
2. Broad experience (>5 years): Years of experience in managing intravitreal treatments for nAMD/DME higher than the median time obtained in the study sample.

### 2.4. Sample Size and Statistical Analysis

Sample size was estimated based on the minimum number of subjects needed to set the exercise of preferences, considering that eight theoretical scenarios would be presented to the HPs. Since a regression model would be built with their answers, a minimum of ten observations per scenario was required (i.e., 80 HP). To ensure the expected minimum sample of 80 complete questionnaires, a participation of 100 HPs was estimated. Finally, the valid participation of 91 HPs was obtained, thus exceeding the minimum necessary sample.

Descriptive statistics were presented for all variables. Continuous variables were analyzed with measures of central tendency (mean and median), variability/dispersion (standard deviation [SD], interquartile ranges, and minimum and maximum), and categorical variables with distributions of absolute and relative frequencies (percentages of groups).

All statistical hypotheses were contrasted against bilateral alternatives and with a significance level of 5%. No adjustments for multiple comparisons or multiple analyses were performed. The statistical analysis was performed using SPSS version 19.0 and Statistical Analysis Software (SAS) Enterprise Guide version 7.15. SPSS uses the algorithm OLS to estimate profits.

## 3. Results

A total of 118 HPs were registered: 98 completed the selection criteria and 91 completed the two preferences exercises. The mean (SD) age was 39.2 (10.2) years, 60.4% were female, and the mean (SD) time of experience as a HP was 12.6 (8.3) years, with a median of 10 years. Only in two cases (2.2%) was the experience < 1 year. A total of 83 (91.2%) HPs stated more than one year of experience in managing intravitreal treatments, with 7.4 (5.0) years being their mean time of experience. The main characteristics of the study population are shown in Table 1.

### 3.1. HPs’ Preferences for the Different Attributes of Intravitreal Treatments

**Figure 1:**
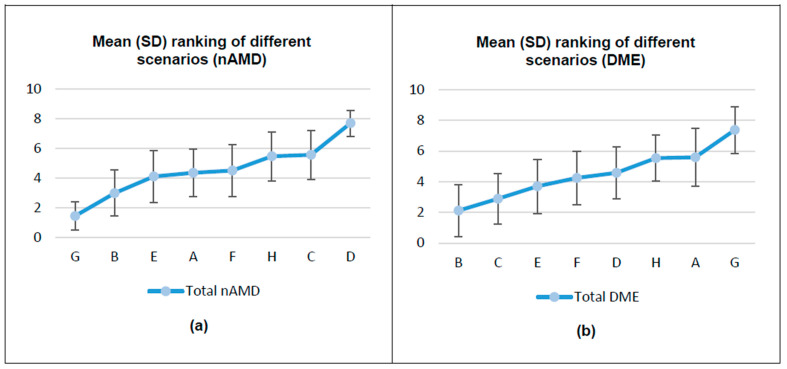
Mean (SD) ranking of different scenarios: (**a**) nAMD; (**b**) DME. DME, diabetic macular edema; nAMD, neovascular age-related macular degeneration; SD, standard deviation.

In the nAMD exercise, scenario G (improvement ≥ 15 letters, mild but more frequent AE, no effect/decrease annual drug cost, prefilled syringe presentation, and MoA with less spacing between doses) was the most preferred treatment scenario (76.9% of HPs selected this scenario as the most preferred one). In contrast, the least preferred (85.7% of HPs) was scenario D (improvement < 15 letters, severe but less frequent AE, annual drug cost increase, vial presentation, and MoA with less spacing between doses) (Figure 1).

In the DME exercise, the most preferred treatment scenario (58.2% of HPs) was scenario B (improvement ≥ 15 letters, mild but more frequent AE, no effect/decrease annual drug cost, vial presentation, and MoA with more spacing doses). In contrast, scenario G (improvement < 15 letters, severe but less frequent AE, annual drug cost increase, vial presentation, and MoA with less spacing between doses) was the least preferred (80.2% of HPs selected them as the least preferred) (Figure 1).

### 3.2. Utility Scores and Importance

Table 2 describes the estimated utilities reported by HPs for attributes and levels assessed in hypothetical treatment scenarios. In both exercises, HPs showed a greater preference for treatment scenarios with high effectiveness in terms of BCVA, no effect on annual drug cost, more spacing between doses, and treatments with frequent mild AEs rather than infrequent serious AEs. In terms of treatment presentations, HPs showed a greater preference for prefilled syringes (Table 2). Utilities obtained were consistent with the information collected at each attribute level, where desired levels of attributes offer greater utilities. Pearson’s R and Kendall’s τ coefficients showed high correlations: between 0.998 (*p* < 0.001) and 1.000 (*p* < 0.001) for nAMD and 0.991 (*p* < 0.001) and 1.000 for DME, respectively.

For intravitreal treatments for nAMD, participants placed the greatest relative importance on BCVA (38.6%), followed by ocular AE (27.3%), annual drug cost (16.3%), available presentation (11.1%), and MoA (6.7%) (Table 2). The importance assigned to each attribute showed minor differences according to the method used (average or relative importance).

For the DME exercise, according to relative importance, the most important attribute for an intravitreal treatment was BCVA (44.6%), followed by ocular AE (25.5%), annual drug cost (17.9%), available presentation (7.3%), and MoA (4.8%). Average importance, obtained at the HP level, was similar but not equal in order in the last attributes: BCVA (34.5%), followed by ocular AE (25.1%), available presentation (15.5%), annual drug cost (15.4%), and MoA (9.5%) (Table 2).

### 3.3. HPs’ Preferences According to Clinical Experience

**Figure 2:**
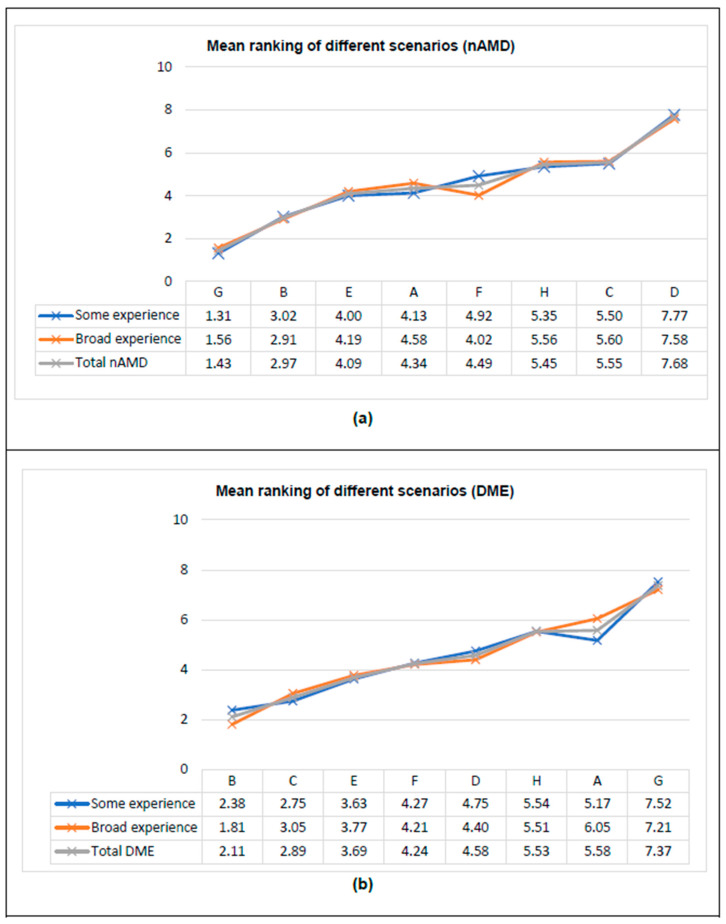
Mean ranking of different scenarios by clinical experience: (**a**) nAMD; (**b**) DME. DME, diabetic macular edema; nAMD, neovascular age-related macular degeneration.

HPs were stratified and analyzed into two different groups according to their previous experience in managing intravitreal treatments for nAMD/DME: 48 HPs with experience of ≤5 years (some experience; 52.7%) and 43 with ≥5 years of experience (broad experience; 47.3%). Higher and lower rankings were similar in both groups. In the nAMD exercise, a difference in ranking was observed in scenario F, while in the DME exercise, it was observed in scenarios A and B (Figure 2).

As previously observed in the global analysis, in the nAMD exercise, scenario G was the most preferred by 83.3% of HPs with some experience and by 69.8% of HPs with broad experience, while scenario D was the least preferred in the two groups (91.7% and 79.1%, respectively). Likewise, in the DME exercise, scenario B was the most preferred by 45.8% of HPs with some experience and by 72.1% of HPs with broad experience, while scenario G was the least preferred in the two groups (83.3% and 76.7%, respectively).

Both groups showed a greater preference for treatment scenarios with high efficacy and safety in terms of BCVA (≥15 letters) and mild frequent AEs, in addition to no increase in cost. In terms of treatment administrations for nAMD, HPs showed a greater preference for prefilled syringes and more spacing between doses. For DME, HPs with some experience preferred prefilled syringes followed by an implant and vial, while HPs with broad experience preferred prefilled syringes followed by a vial and implant (Table 3).

In the nAMD exercise, HPs with some experience managing intravitreal treatments showed a greater preference for treatments with higher BCVA effectiveness (relative importance: 41.1%). Furthermore, they placed greater importance on ocular AE (25.2%), annual drug cost (15.9%), and available presentation (13.6%), while giving lower importance to MoA (4.2%). On the other hand, HPs with broad experience showed a greater preference for treatments with higher BCVA effectiveness (35.8%), greater importance on ocular AE (29.7%), and annual drug cost (16.8%), as well as similar importance between MoA (9.6%) and available presentation (8.2%) (Table 3).

In the DME exercise, the order of importance was the same among HPs with some experience and broad experience, although the group with broad experience assigned a similar importance to available presentation and MoA, giving greater importance to effectiveness and AE (Table 3).

## 4. Discussion

nAMD and DME are two chronic diseases that can progress and lead to blindness within a few years [8,9,11,12,13,26]. Although current treatments make it possible to delay the progression of these diseases and preserve existing vision, there is a growing interest in considering the added value of new therapies, not only based on effectiveness, but also on other characteristics that may be relevant for the different stakeholders [14,16,17,26]. Differences in BCVA, ocular AE, annual drug cost, available presentation, and MoA lead to uncertainty among HPs who manage the intravitreal treatments based on their own experience and patients’ preferences.

Decision-making in the selection of a treatment or health intervention is a complex process due to the presence of multiple factors and differing objectives that can influence the optimal decision [17]. This process becomes even more complicated when the opinions of different stakeholders and patient perspectives are considered. Understanding the preferences of each group, even when they may differ, can be crucial in the decision-making process [16,17,18].

Although there are different estimation procedures in the application of CA techniques to assess preferences [27], OLS regression was selected in the present study to estimate parameters, as it presents major advantages because it produces results that are virtually identical to those obtainable with non-metric procedures, which are more complex from computational point of view.

The HP collective in Spain has a growing interest in evaluating patients’ outcomes as the most important consequence of their professional activity, although there are a lack of publications evaluating their treatment preferences in Spain. Only two recent studies were identified: a preferences study in the field of multiple sclerosis [18] and a multicriteria decision analysis (MCDA) for DME treatments with multiple stakeholders, including pharmacists [17]. Martínez-López et al. concluded that understanding which treatment characteristics are meaningful to HPs may help to enhance their synergistic role in the multidisciplinary management of patients with multiple sclerosis, although further studies are necessary to gather a more comprehensive spectrum of HP-relevant attributes [18]. De Andrés-Nogales et al. conducted a MCDA to determine the most relevant criteria to decision-making in the management of DME based on the perspectives of multiple stakeholders in Spain, concluding that, from a multi-stakeholder perspective, the selection of an appropriate DME treatment should guarantee patient safety and maximize visual acuity improvement and treatment effect duration, also contributing to system sustainability by being affordable, having a positive impact on health-related quality of life, and preventing disability [17].

Our study shows that HPs placed the highest relative importance on BCVA, followed by ocular AE, annual drug cost, available presentation, and MoA, for both exercises, nAMD and DME, with the same conclusion regarding the average importance except for DME exercise, where HP prioritized available presentation over annual drug cost. So, as in other therapeutic areas, such as multiple sclerosis [18], effectiveness and safety are the most relevant attributes.

Only small differences were found between HPs with some versus broad experience in managing intravitreal treatments for nAMD/DME. In the case of nAMD, differences were observed for scenarios A and G, and, in DME, for scenarios A and B. Both groups (some and broad experience managing intravitreal treatments) showed a greater preference for treatment scenarios with high efficacy and safety in terms of BCVA (≥15 letters) and mild frequent AEs, in addition to no increase in cost. In terms of treatment administrations for nAMD, HPs with some experience showed a greater preference for prefilled syringes, and those with broad experience preferred more spacing between doses. For DME, HPs with some experience preferred prefilled syringes followed by implants and vials, while those with broad experience preferred prefilled syringes followed by vials and implants.

There were a few limitations that should be considered while interpreting the outcomes. One possible limitation is inherent to preference elicitation studies, as the preference weights obtained are specific to the attributes and levels of each attribute that are presented, and it is not feasible to include all the attributes and levels that may influence the preferences. A large number of attributes and levels can increase the cognitive difficulty of choice and rank the scenarios, but the inclusion of fewer attributes can omit key attributes [28]. However, the study design phase was conducted to identify and select the final attributes and levels to design the scenarios, and much emphasis was placed on the panel of experts meeting to select only those attributes that they considered most relevant, trying to minimize the number of levels in each one. The second limitation is that, although approximately 2000 HPs experienced in managing intravitreal treatments for nAMD/DME were invited to participate, only 91 (4.6%) who finally accept to participate were considered valid for the present analysis by meeting the selection criteria and having completed the two preference exercises. In any case, the final number of participants is over the minimum needed sample size (80 HPs). Also, this was an exploratory study, and no extrapolation to the total population was planned to be made. Finally, results obtained in the current study must be interpreted considering all attributes and levels used to define scenarios. The importance assigned to each attribute corresponds to relative importance, which is defined according to the importance assigned to one attribute in comparison with all other attributes. Based on that, the importance of specific attributes cannot be interpreted or compared with other studies independently of other attributes used in the study.

## 5. Conclusions

This study describes the use of CA to evaluate HPs’ preferences for different hypothetical intravitreal treatments with anti-VEGF or corticosteroids for nAMD and DME, according to a set of selected attributes (BCVA, ocular AE, annual drug cost, available presentation, and MoA), and aims to quantify the weight of each studied attribute. Effectiveness and safety were the most important attributes, while cost, available presentation, and MoA were not as important for the HPs when choosing a drug, although “no effect/decrease annual drug cost” was included in the most preferred treatment scenario for both diseases. These preferences were comparable independent of the experience of the HP, although some differences according to the type of hospital and the number of nAMD/DME treatments were observed for the importance given to the available presentation. Understanding which treatment characteristics are meaningful to HPs may help to enhance their synergistic role in the multidisciplinary management of patients with nAMD and DME. Further studies are needed to explore a more comprehensive spectrum of HP-relevant attributes.

## Figures and Tables

**Table 1:** Demographic and professional practice characteristics of the sample.

Characteristics	Total Sample (*n* = 91) *
Age, years, mean (SD)	39.2 (10.2)
Sex, female, *n* (%)	55 (60.4%)
Years as a HP, mean (SD) (valid *n* = 89)	12.6 (8.3)
Years of managing patients with intravitreal treatments for nAMD/DME, *n* (%)	
<1 year	8 (8.8%)
≥1 year	83 (91.2%)
Number of years of experience managing intravitreal treatments, mean (SD) (valid *n* = 83)	7.4 (5.0)
Level of clinical experience according to years of experience managing intravitreal treatments (quartile definition)	
Some experience (≤5 years)	48 (52.7%)
Broad experience (>5 years)	43 (47.3%)
Participation in nAMD/DME clinical trials, *n* (%)	12 (13.2%)
Author or co-author of scientific publications, *n* (%)	44 (48.4%)
Participation in a pharmacy commission for the evaluation of medicines, *n* (%)	73 (80.2%)
Current position	
Head of service	16 (17.6%)
Specialist pharmacist	63 (69.2%)
Pharmacy resident	12 (13.2%)
Pharmacist profile, *n* (%)	
Manufacturer/sterile area	44 (48.4%)
Logistic area	47 (51.6%)
Type of hospital, *n* (%)	
Regional hospital	25 (27.5%)
University hospital	66 (72.5%)
Number of treatments prepared for intravitreal administration in a month, mean (SD)	
For nAMD (valid *n* = 87)	215.7 (232.8)
For DME (valid *n* = 86)	76.0 (94.2)

* Unless otherwise specified. DME, diabetic macular edema; HP, hospital pharmacist; nAMD, neovascular age-related macular degeneration; SD, standard deviation.

**Table 2:** Utility scores and importance assigned to each attribute and level.

Variable		Utility Estimation		nAMD (*n* = 91)		DME (*n* = 91)	
		nAMD (*n* = 91)	DME (*n* = 91)	Importance (Relative)	Importance (Averaged)	Importance (Relative)	Importance (Averaged)
BCVA	≥15 letters	1.294	1.266	38.6%	36.5%	44.6%	34.5%
	<15 letters	−1.294	−1.266				
Ocular AE	Severe but less frequent AE	−0.915	−0.723	27.3%	27.7%	25.5%	25.1%
	Mild but more frequent AE	0.915	0.723				
Annual drug cost	Increase	−0.547	−0.508	16.3%	15.3%	17.9%	15.4%
	No effect/decrease	0.547	0.508				
Available presentation	Prefilled syringe	0.371	0.234	11.1%	13.3%	7.3%	15.5%
	Vial	−0.371	−0.183				
	Implant	NA	−0.051				
MoA	MoA with more spacing between doses	0.225	0.135	6.7%	7.3%	4.8%	9.5%
	MoA with less spacing between doses	−0.225	−0.135				

AE, adverse event; BCVA, best corrected visual acuity; DME, diabetic macular edema; MoA, mechanism of action; nAMD, neovascular age-related macular degeneration.

**Table 3:** Utility scores and importance assigned to each attribute and level by clinical experience.

Variable		Utility Estimation		Importance (Relative)		Importance (Averaged)		
		Some Experience (*n* = 48)	Broad Experience (*n* = 43)	Some Experience (*n* = 48)	Broad Experience (*n* = 43)	Some Experience (*n* = 48)	Broad Experience (*n* = 43)	*p*-Value
**nAMD**								
BCVA	≥15 letters	1.385	1.192	41.1%	35.8%	38.5%	34.3%	0.2826
	<15 letters	−1.385	−1.192					
Ocular AE	Severe but less frequent AE	−0.849	−0.988	25.2%	29.7%	25.8%	29.8%	0.3132
	Mild but more frequent AE	0.849	0.988					
Annual drug cost	Increase	−0.536	−0.558	15.9%	16.8%	14.7%	15.9%	0.6368
	No effect/decrease	0.536	0.558					
Available presentation	Prefilled syringe	0.458	0.273	13.6%	8.2%	15.5%	10.8%	0.1169
	Vial	−0.458	−0.273					
MoA	MoA with more spacing between doses	0.141	0.320	4.2%	9.6%	5.5%	9.3%	0.0253
	MoA with less spacing between doses	−0.141	−0.320					
**DME**								
BCVA	≥15 letters	1.245	1.291	42.5%	46.0%	33.0%	36.2%	0.4000
	<15 letters	−1.245	−1.291					
Ocular AE	Severe but less frequent AE	−0.646	−0.808	22.1%	28.8%	24.3%	26.0%	0.6592
	Mild but more frequent AE	0.646	0.808					
Annual drug cost	Increase	−0.521	−0.494	17.8%	17.6%	16.5%	14.2%	0.3891
	No effect/decrease	0.521	0.494					
Available presentation	Prefilled syringe	0.354	0.101	12.2%	3.8%	16.4%	14.5%	0.5565
	Vial	−0.359	0.014					
	Implant	0.005	−0.114					
MoA	MoA with more spacing between doses	0.161	0.105	5.5%	3.7%	9.8%	9.2%	0.7549
	MoA with less spacing between doses	−0.161	−0.105					

AE, adverse event; BCVA, best corrected visual acuity; DME, diabetic macular edema; MoA, mechanism of action; nAMD, neovascular age-related macular degeneration.

## Data Availability

The data that support the findings of this study are available from the corresponding author upon reasonable request.

## Supplementary Materials

The following supporting information can be downloaded at: https://www.mdpi.com/article/10.3390/pharmacy13030068/s1, Table S1: Attributes and levels that configure the scenarios; Table S2: Set of preferences scenarios.

## Abbreviations

The following abbreviations are used in this manuscript:

AE	Adverse events
BCVA	Best corrected visual acuity
CA	Conjoint analysis
DME	Diabetic macular edema
HP	Hospital pharmacist
MoA	Mechanism of action
nAMD	Neovascular age-related macular degeneration
SD	Standard deviation
