# Supplementary material for: Preferences of Hospital Pharmacists for the Different Attributes of Intravitreal Treatments for Neovascular Age-Related Macular Degeneration and Diabetic Macular Edema in Spain: The SEEKING Study
Source: Pharmacy (Basel). 2025 May 14;13(3):68. doi: 10.3390/pharmacy13030068 (PMC12101423; doi:10.3390/pharmacy13030068)
Supplement: Supplementary file 1 [file pharmacy-13-00068-s001.zip › pharmacy-3601751-supplementary.pdf]

Supplementary Table S1. Attributes and levels that configure the scenarios.

Selected attributes	Definition	Selected levels: nAMD	Selected levels: DME
BCVA	It is one of the most widely used methods for quantifying visual acuity, especially in clinical trials (primary objective) of intravitreal therapies for nAMD/DME treatment.<br>It was assessed through the ETDRS letters improvement from baseline.<br>The improvement proposed in the TENAYA trial (15 letters as cut-off point) was used.	• ≥15 letters<br>• <15 letters	• ≥15 letters<br>• <15 letters
Ocular AE*	In most phase 3 clinical trials with intravitreal treatments, both ocular and non-ocular (systemic) AE are evaluated, but the former plays a vital role.<br>Some of the most frequent ocular AE are: endophthalmitis (most frequent AE), cataracts, conjunctival hemorrhage, and retinal tear.<br>Other AE such as intraocular inflammation require a special follow-up of the patients.	• Severe but less frequent AE<br>• Mild but more frequent AE	• Severe but less frequent AE<br>• Mild but more frequent AE
Annual drug cost	It is one of the key factors in the intravitreal treatment decision.<br>For the assessment, the unit cost of the drug, the number of injections per year, or both, were considered.	• Increase<br>• No effect/decrease	• Increase<br>• No effect/decrease
Available presentation	There is a wide variety of presentations available for intravitreal treatments: syringes, vials, implant, etc.<br>They influence the need for treatment management by the hospital pharmacy (logistic/administrative area and drug preparation).	• Prefilled syringe<br>• Vial	• Prefilled syringe<br>• Vial<br>• Implant
MoA	There are two groups of intravitreal treatments available, according to the MoA: corticosteroids (only for DME) and anti-VEGF.<br>The MoA can be related to the durability of the treatment (interval or time between injections). The current trend is that new treatments, with newer MoA, achieve better dosing intervals (longer time between injections).	• MoA with more spacing between doses<br>• MoA with less spacing between doses	• MoA with more spacing between doses<br>• MoA with less spacing between doses

*Mild AE: discomfort noticed, but no disruption of normal daily activity. Severe AE: incapacitating with inability to work or to perform normal daily activity.
AE, adverse event; BCVA, best corrected visual acuity; DME, diabetic macular edema; ETDRS, early treatment diabetic retinopathy study; MoA, mechanism of action; nAMD, neovascular age-related macular degeneration; VEGF, vascular endothelial growth factor.

Supplementary Table S2. Set of preferences scenarios.

	A	B	C	D	E	F	G	H
**nAMD**								
BCVA	≥15 letters	≥15 letters	<15 letters	<15 letters	≥15 letters	<15 letters	≥15 letters	<15 letters
Ocular AE*	Severe but less frequent AE	Mild but more frequent AE	Severe but less frequent AE	Severe but less frequent AE	Severe but less frequent AE	Mild but more frequent AE	Mild but more frequent AE	Mild but more frequent AE
Annual drug cost	No effect/decrease	Increase	No effect/decrease	Increase	Increase	No effect/decrease	No effect/decrease	Increase
Available presentation	Vial	Vial	Prefilled syringe	Vial	Prefilled syringe	Vial	Prefilled syringe	Prefilled syringe
MoA	Less spacing between doses	More spacing between doses	More spacing between doses	Less spacing between doses	More spacing between doses	More spacing between doses	Less spacing between doses	Less spacing between doses
**DME**								
BCVA	<15 letters	≥15 letters	≥15 letters	<15 letters	≥15 letters	≥15 letters	<15 letters	<15 letters
Ocular AE*	Severe but less frequent AE	Mild but more frequent AE	Mild but more frequent AE	Mild but more frequent AE	Severe but less frequent AE	Severe but less frequent AE	Severe but less frequent AE	Mild but more frequent AE
Annual drug cost	No effect/decrease	No effect/decrease	Increase	No effect/decrease	No effect/decrease	Increase	Increase	Increase
Available presentation	Prefilled syringe	Vial	Prefilled syringe	Prefilled syringe	Implant	Prefilled syringe	Vial	Implant
MoA	More spacing between doses	More spacing between doses	Less spacing between doses	Less spacing between doses	Less spacing between doses	More spacing between doses	Less spacing between doses	More spacing between doses

*Mild AE: discomfort noticed, but no disruption of normal daily activity. Severe AE: incapacitating with inability to work or to perform normal daily activity.

AE, adverse event; BCVA, best corrected visual acuity; DME, diabetic macular edema; MoA, mechanism of action; nAMD, neovascular age-related macular degeneration.
